# Geospatial characteristics of non-motor vehicle and assault-related trauma events in greater Phoenix, Arizona

**DOI:** 10.1186/s40621-020-00258-x

**Published:** 2020-06-15

**Authors:** Alan Cook, Robin Harris, Heidi E. Brown, Edward Bedrick

**Affiliations:** 1 Department of Epidemiology and Biostatistics, University of Texas Health Science Center Tyler School of Community and Rural Health, 11937 U.S. Highway 271, H252, Tyler, TX 75708 USA; 2 Department of Epidemiology and Biostatistics, University of Arizona Mel and Enid Zuckerman College of Public Health, 1295 N. Martin Ave., Drachman Hall, Tucson, AZ 85724 USA

**Keywords:** Trauma, Injury, Assault, Socioeconomic status, Geospatial, Geostatistical

## Abstract

**Background:**

Injury-causing events are not randomly distributed across a landscape, but how they are associated with the features and characteristics of the places where they occur in Arizona (AZ) remains understudied. Clustering of trauma events and associations with areal sociodemographic characteristics in the greater Phoenix (PHX), AZ region can promote understanding and inform efforts to ameliorate a leading cause of death and disability for Arizonans. The outcomes of interest are trauma events unrelated to motor vehicle crashes (MVC) and the subgroup of trauma events due to interpersonal assaults.

**Methods:**

A retrospective, ecological study was performed incorporating data from state and national sources for the years 2013–2017. Geographically weighted regression models explored associations between the rates of non-MVC trauma events (n/10,000 population) and the subgroup of assaultive trauma events per 1000 and areal characteristics of socioeconomic deprivation (areal deprivation index [ADI]), the density of retail alcohol outlets for offsite consumption, while controlling for race/ethnicity, population density, and the percentage urban population.

**Results:**

The 63,451 non-MVC traumas within a 3761 mile^2^ study area encompassing PHX and 22 surrounding communities, an area with nearly 60% of the state’s population and 54% of the trauma events in the AZ State Trauma Registry for the years 2013–2017. Adjusting for confounders, ADI was associated with the rates of non-MVC and assaultive traumas in all census block groups studied (mean coefficients 0.05 sd. 0.001 and 0.07 sd. 0.002 for non-MVC and assaultive trauma, respectively). Alcohol retail outlet density was also associated with non-MVC and assaultive traumas in fewer block groups compared to ADI.

**Conclusion:**

Socioeconomic deprivation and alcohol outlet density were associated with injury producing events in the greater PHX area. These features persist in the environment before and after the traumas occur. Ongoing research is warranted to identify the most influential areal predictors of traumatic injury-causing events in the greater PHX area to inform and geographically target prevention initiatives.

## Introduction

Traumatic injuries affect individuals, families and communities. Nationally, unintentional injury is the leading cause of death and disability for Americans one to 44 years-old and the third leading cause of death overall, primarily due to brain injuries.(Centers for Disease Control and Prevention National Center for Injury Prevention and Control, 2010; Gunst et al., 2010) According to the most recent report of the National Vital Statistics System from the Centers for Disease Control and Prevention, in 2016 over 161,000 people in the United States (US) died from unintentional injuries. Intentional self-harm (suicide) was responsible for 45,000 deaths. Motor vehicle crashes and falls combined for another 75,000 deaths.(Xu et al., n.d.) In Arizona (AZ), 46,842 patients were treated for traumatic injuries, of whom 1111 (2.4%) people (75 per 100,000) died in 2016.(Arizona Bureau of Emergency Medical Services and Trauma System, 2017) In addition to the toll of lives lost, an enormous financial cost is associated with fatal and nonfatal injury. The annual inpatient costs for trauma patients in the US have been estimated to be more than $37 billion dollars.(Velopulos et al., 2013) When lifetime productivity losses are included, the costs exceed $99 billion dollars among those involved in fatal and nonfatal motor vehicle crashes.(Naumann et al., 2010) In 2016, the sum of trauma center charges in AZ was $1.9 billion with a median charge of $22,418 per patient.(Arizona Bureau of Emergency Medical Services and Trauma System, 2017)

Traumatic injury is multifactorial and is the results of unintentional or intentional actions. Certain environmental features associated with traumatic injury-causing events have been identified from the sociology, medicine, criminal justice, and public health disciplines. The availability of alcohol bears a strong and consistent association with traumatic injury, particularly assault-related mechanisms, whether measured among individuals in the emergency department or at the community level.(Sheppard et al., 2008; Pridemore & Grubesic, 2013; Britt et al., 2005; Cameron et al., 2016; Cunradi et al., 2011; Cunradi et al., 2012; Gorman et al., 1998; Gorman et al., 2005; Grubesic & Pridemore, 2011; Lipton & Gruenewald, 2002; Livingston, 2011; Reid et al., 2003; Resko et al., 2010; Scribner et al., 1995; Zhu et al., 2004) Similarly, neighborhood social disadvantage or socioeconomic status (SES) has been associated with interpersonal violence and injury.(Bouffard & Muftić, 2006; Boyle & Hassett-Walker, 2008; Valdez et al., 2007; Simpson et al., 2005; Abedzadeh-Kalahroudi et al., 2018; Chong et al., 2015; Halonen et al., 2012; Jarman et al., 2018a; Lawson et al., 2015; Markowitz, 2003; Zarzur et al., 2010) Numerous metrics of areal SES have been evaluated by previous investigators for association with traumatic injury and survival.(Bouffard & Muftić, 2006; Boyle & Hassett-Walker, 2008; Valdez et al., 2007; Simpson et al., 2005; Abedzadeh-Kalahroudi et al., 2018; Chong et al., 2015; Halonen et al., 2012; Jarman et al., 2018a; Lawson et al., 2015; Bell et al., 2015; Cubbin et al., 2000a; Cubbin & Smith, 2002; Jarman et al., 2018b) However, no consensus regarding the best features to model SES has been established.(Kruithof et al., 2017)

It is unlikely that alcohol availability and SES promote injury-causing events uniformly across geographic regions and populations. Arizona has several unique characteristics which may modify the associations of areal sociodemographic features with the occurrence and locations of trauma. These distinctive features of the population include the proportion living below the poverty level and the racial and ethnic composition living in AZ.(United States Census Bureau, 2015) As such the sociodemographic features of AZ may interact with factors known to be related to the occurrence of injury-related events in ways distinct from other regions. Moreover, the features considered as predictive variables under study are considered to be neither uniformly present nor randomly distributed in geographic terms. Therefore, the geographically varying concentrations of such predictors may influence the occurrence of trauma events in similarly varying degrees and are the focus of this study. No prior study has undertaken the characterization of the geographic associations of trauma in AZ with the published predictors of traumatic injury events, specifically SES and the availability of alcohol via retail outlets for off-site consumption.

**Fig. 1:**
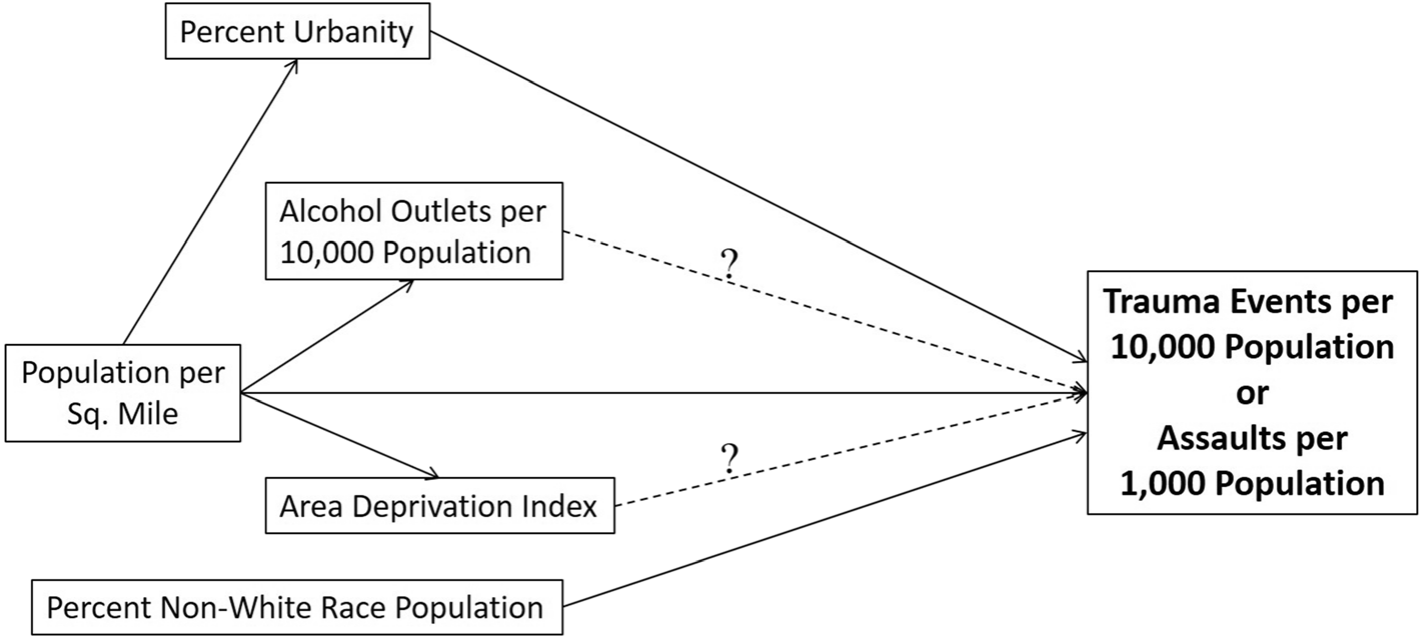
Hypothesized causal pathway linking study predictors and outcomes

Traumatic injury-causing events are not randomly distributed across a landscape, but how they are associated with the features and characteristics of the places where they occur in AZ remains understudied. Elucidating the clustering of trauma events and associations with the areal features of population density, racial and ethnic composition, proportion urban population, socioeconomic deprivation, and alcohol availability in the greater Phoenix (PHX), AZ region can promote understanding of the mosaic of environmental promoters of injurious events and inform efforts to ameliorate a leading cause of death and disability for Arizonans. The goal of this study was to geographically model the distributions of traumatic injury-causing events and hypothesized predictors. Specifically, we hypothesized that socioeconomic deprivation and alcohol availability would be positively associated with non-motor vehicle crash (MVC) trauma events and a subgroup of trauma events arising from interpersonal assault leading to injury sufficiently severe to warrant evaluation and treatment in emergency department of a hospital in PHX. The hypothesized causal pathway linking socioeconomic deprivation and alcohol outlet density to non-MVC trauma events and assault-related trauma events controlling for the confounding effects of population density, percent urban population, and percentage of non-White race population is depicted in Fig. 1.

## Methods

This is a retrospective, ecological study using geospatial and aspatial analysis of trauma in AZ for the years 2013–2017. It was approved by the human subjects review boards of the University of Arizona (Protocol Number: 1810983290) and the AZ Department of Health Services (AZDHS) (HSRB 18–0047).

Patients were included if they met AZ State Trauma Registry (ASTR) of the AZDHS inclusion criteria. Briefly, patients are included in the ASTR registry if the emergency medical team transporting the patient deems the patient has suffered traumatic injury, transferred from one trauma center to another via EMS, required a trauma team activation, or were admitted to hospital or died due to traumatic injuries.(Arizona Department of Health Services Emergency Medical Services and Trauma System, 2019) Patients were excluded from this study if their trauma occurred outside of AZ, their injuries were caused by burns, drowning, insect or animal bites or stings, overexertion, poisoning, or other mechanisms unrelated to gross anatomic injury. Patients were also excluded from the analysis if they were injured as a driver or passenger involved in a MVC. This exclusion is based the distribution of motor vehicle safety features favoring upper-income drivers and places individuals from lower SES at increased risk of harm.(Girasek & Taylor, 2010) This disparity would create a differential risk of requiring treatment for injuries due to MVC leading to a differential representation in the data. Moreover, analysis of data from the South Carolina Department of Motor Vehicles shows only 35% of MVCs happen within five miles of the drivers’ residential address.(Brown, 2016) Thus, the inclusion of MVCs in the present study would systematically bias the results. Burns were excluded as the mechanism of injury differs significantly from the others included in the study. Additionally, there is a single adult burn center and a single pediatric burn center in AZ. Patients were also excluded if their survival status was not known or if no address of the incident was included in their records.

Patient-level trauma data from the ASTR included patient age group, sex, race and ethnicity, comorbid diseases, location of trauma incident, type of insurance, mechanism of injury, clinical diagnoses for injuries, vital status at hospital discharge (alive/dead), and hospital discharge destination. Injury diagnoses were described in the Abbreviated Injury Scale (AIS),(Gennarelli & Wodzin, 2008) International Classification of Diseases, Ninth Revision, Clinical Modification, (ICD-9)(World Health Organization, National Center for Health Statistics, Centers for Medicare and Medicaid Services, 2010) and the International Classification of Diseases, Tenth Revision, Clinical Modification, (ICD-10).(World Health Organization, National Center for Health Statistics, Centers for Medicare and Medicaid Services, 2015) The severity of traumatic injury of each individual is a critical factor when aggregating and comparing groups of people with diverse injury mechanisms and diagnoses. Injury severity was quantified using the Trauma Mortality Prediction Model probability of death (TMPM).(Osler et al., 2019; Osler et al., 2008; Glance et al., 2009) Events identified as assault-related were defined according to the External Causes of Injury and Poisoning manner and intent of injury for both the ICD-9-CM and ICD-10-CM lexicons in the AZDHS files.

**Fig. 2:**
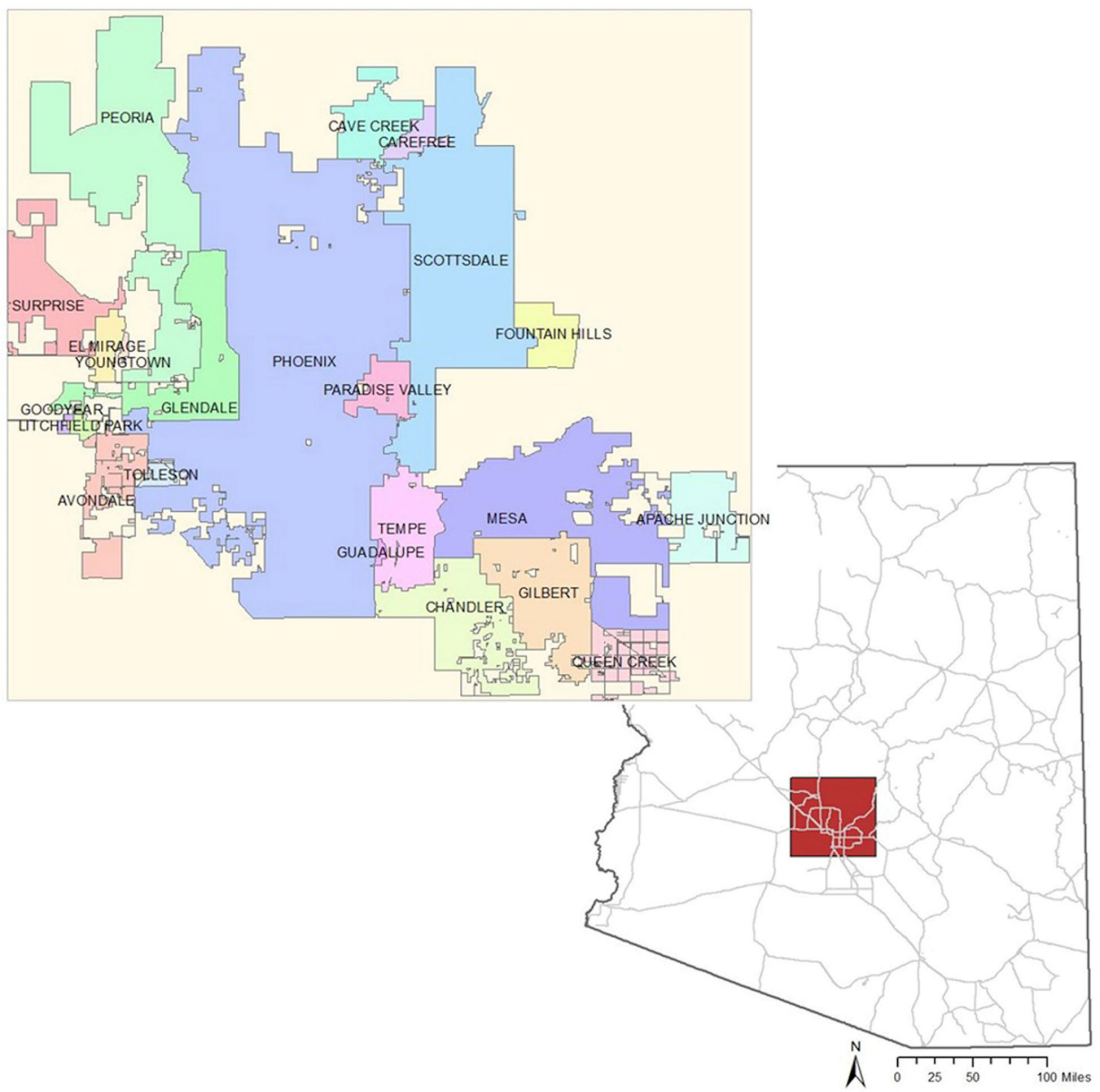
Greater Phoenix study area with incorporated municipalities

The study region of interest was a 3,761 mile^2^ rectangular area incorporating the city limits of Phoenix, as well as 22 surrounding communities. This approach was chosen to focus the first analysis to characterize the geographic associations of traumatic injury events and published predictors of such events to an area that captures a majority of the state’s population with fewer zero-population block groups (2 in study area versus 21 for entire state) Fig. 2.

Numerous metrics of areal SES have been evaluated by previous investigators for association with traumatic injury and survival. However, no consensus regarding the best construct has been established.(Bell et al., 2015; Cubbin et al., 2000a; Cubbin & Smith, 2002; Jarman et al., 2018b; Kruithof et al., 2017; Cubbin et al., 2000b; Cubbin et al., 2000c; Jarman et al., 2016; Kennedy et al., 1998; Marcin et al., 2003; Messer et al., 2006; Shenassa et al., 2004) For example, Bell, et al., conducted a review of 33 studies and identified 70 census-based indicators for monitoring areal risks of trauma.(Bell et al., 2015) These included level of education and employment-related measures. Cubbin, et al., found housing value, crowded housing and blue collar occupation are associated with injury risk.(Cubbin et al., 2000b) To examine the association of socioeconomic deprivation and the scene of traumatic injury, we chose the Areal Deprivation Index (ADI) as the metric of SES.(Singh, 2003; Knighton et al., 2016) Shapefiles for US Census block groups were downloaded from the US Census Bureau’s TIGER/Line Shapefiles.(United States Census Bureau, 2018a) Block group demographics and information pertaining to income, poverty, housing, employment, and education were obtained from the American Communities Survey and Decennial Census from the American FactFinder website.(United States Census Bureau, 2010; United States Census Bureau, 2013) The ADI is calculated by summing the 17 data points for each census block-group, weighted by their Singh coefficient thus producing a base score.(Singh, 2003) The coefficients range from ^−^ 0.0823 to 0.1157. Next, the ADI is standardized and computed so the resulting mean for all block groups equaled 100 with a standard deviation of 20. An alternative measure of the dispersion of wealth among the block groups in the study area, the Gini index or Gini coefficient, was compared to the ADI in the geographic models of this study. The Gini index is a common measure of the dispersion of wealth among members of a group and theoretically ranges from 0 to 1. A Gini coefficient of 0 represents perfect equality among the group members while a coefficient of 1 indicates a single member of the group holds all of the wealth. The models were run with ADI, with the Gini coefficient and no measure of SES. The model with ADI produced the lowest Akaike Information Criterion of the three with no difference in the R^2^ coefficient of determination. In addition, the *p*-values for the Gini index were greater than 0.05, whereas the p-values for ADI were significant. Thus, the ADI was chosen as the measure of SES for the study.

The alcohol license type and addresses were downloaded from the Alcohol License Master Table from the Arizona Department of Liquor website.(Arizona Department of Liquor, 2019) Liquor stores and beer and wine stores, including those with sampling privileges were included as alcohol outlets for offsite consumption. Retail outlet density per 10,000 population was computed as the number of outlets in a block group divided by the respective total population and rescaled by a factor of 10,000.

Data preparation began with geocoding the addresses of trauma scenes and alcohol outlets. This was accomplished using the Geocode Addresses tool within ArcGIS Desktop, version 10.6.1 command with Street_Addresses_US as the locator. Addresses unrecognized by the algorithm were geocoded by hand using Google Map.

The geospatial unit of analysis was the census block group. The block groups are arranged to include 600 to 3,000 people. Block groups contain census blocks all within a census tract. Each census tract contains a minimum of one block group.(United States Census Bureau, 2012) The study area can be subdivided into 2,507 census block groups, or 923 census tracts, or 132 Zip Code Tabulation Areas. Census block groups unit were chosen as the unit of analysis for several reasons. First, the published Singh Coefficients for the ADI were estimated at the block group level. Moreover, the trauma events and alcohol outlets are point events which require aggregation to some larger geographic areas. Aggregation of individual (point) SES data to higher geographic units introduces bias with larger regression coefficients compared to the data at the individual level.(Soobader et al., 2001) However, aggregation to comparatively smaller areas introduces less bias.(Soobader et al., 2001) Population density was computed as the quotient of each block group’s total population divided by the area in square miles. These data were assembled by spatially linking and aggregating the locations of non-MVC traumatic injury causing events, including those due to assault, to their respective block group polygon. The racial and ethnic composition of Arizona differs from the US as a whole. For example, the proportion of American Indians in Arizona is 5.3%, which is more than four-fold greater than the remainder of the US (1.3%). Then, the proportion of the population self-identifying as Hispanic/Latino ethnicity is 73% greater in Arizona, compared to the rest of the nation, (31.6% versus 18.3%). In the present study, minority race and ethnicity groups are aggregated and referred to as non-White, which is a commonly applied construct in the trauma literature. For example, areas with higher proportions of non-White residents were shown to be associated with trauma events in a geospatial analysis by Newgard, et al.(Newgard et al., 2011) Finally, although the US Census Bureau’s definition of an urban area includes population density as an element, it also includes paved land coverage, and areas of discontinuous urban development.(Ratcliffe et al., 2016) As such, both were included. The ADI, alcohol retail outlet density, population count, population density, block group area in square miles (sq. mi.), percent urban population (urbanity), and percent non-White population were features within each block group.

First, an exploratory analysis was done to elucidate the global and local measures of autocorrelation between the events of interest, non-MVC traumas and a subgroup of assault-related traumas, and the attributes of ADI, alcohol retail outlet density, percent non-White population, population density, and percent urbanity using Moran’s I statistic(Moran, 1950) and Anselin’s Local Indicators of Spatial Association (LISA)(Anselin, 1995) for global and local, respectively. The Moran’s I is similar to an aspatial correlation coefficient. It varies between − 1.0 and + 1.0 with higher absolute values indicating greater spatial autocorrelation by weighting the values of proximal observations greater than observations far apart. Positive values for the Moran’s I indicate positive autocorrelation. A value of zero demonstrates no clustering of similar values or complete spatial randomness.(Pfeiffer et al., 2008) The LISA analysis can be thought of as a decomposition of the Moran’s I to assess the contributions of each observation and can function to identify areas of nonstationarity.(Anselin, 1995) LISA reports and maps clusters of observations with high values surrounded by other observations with high values (High-High or HH) and clusters of low values surrounded by low values, (Low-Low or LL). The LISA process also reports outliers of high values surrounded by low value observations (High-Low or HL), and conversely low values in local areas of high values (Low-High or LH).

Next, a geographically weighted multivariable regression model was fitted to appraise the strength of association between all injury events and attributes of the places in which they occurred, specifically the density of alcohol retail outlets for off-site consumption within each block group (outlets per 10,000 persons), the ADI, population density (persons per square mile per block group), percent urban population, and the percent non-white population. The same model fitting process was carried out for assaultive trauma events. The rates of non-MVC traumas (events/10,000 population) and assault related trauma events per 1,000 population per block group were the dependent variables in the GWR models, respectively. In both cases the variables were standardized as an initial step in the model fitting process.

The independence of observations is a fundamental assumption underlying aspatial statistical analysis, where the inference of an association is deemed to be constant regardless of where the measurement was taken within the parameter space. Geographic data violate that assumption of independence by virtue of their respective proximity in the geographic region of analysis. Moreover, the phenomenon of interest may not be constant across the study area, which is known as spatial nonstationarity, and must be taken into account in the statistical modeling process. The modeling of nonstationary geographic relationships has been addressed with the development of the geographically-weighted regression (GWR).(Brunsdon et al., 1996; Fotheringham et al., 2003)

Non-spatial data management and analysis were performed using Stata MP version 14.2.(StataCorp, 2015)

Mapping spatial data was accomplished using, and ArcMap 10.6.1.(ESRI, 2011) Moran’s I statistic and Anselin’s LISA were calculated using GeoDa 1.8.(Anselin et al., 2006) GWR regression models were constructed using the MGWR 2.2.1 application (https://sgsup.asu.edu/sparc).(Oshan et al., 2018)

## Results

The state of Arizona covers 113,998 mile^2^ (sq. mi.) with a mean population of 6,364,323 during 2013–2017.(Arizona Office of Economic Opportunity. Population Estimates, 2019) There are 15 counties and 4178 U.S. Census Block Groups within the state. The mean population per block group was 1523.3, (sd 779.4), although 23 block groups have populations of zero. The block groups range in area from 0.03 sq. mi. to 4763.1 sq. mi. resulting in a median population density per block group was 3582.2 people per sq. mi.

Over 177,311 traumatic injury events are present in the ASTR database and 4575 (2.6%) died as a result of those injuries between 2013 and 2017. On average, 35,469 traumas were entered into the ASTR database every year from 2013 to 2017. Table 1 presents characteristics of the 117,253 patients injured by non-MVC mechanisms “study group”. During the five-year study period, the number of traumas in the study area reported to the ASTR increased by 73.1%, from 9247 events in 2013 to 16,008 events in 2017 while the population of the study area increased by only 6.8%.(Arizona Office of Economic Opportunity. Population Estimates, 2019) Therefore, the incidence of non-MVC trauma events meeting criteria for inclusion in ASTR increased by approximately 15.7% per year, from 23.7 cases per 10,000 population in the study area in 2013 to 38.3 per 10,000 in 2017. The trauma patients were more often male, (58.8% versus 41.2%) and the 50–69 year-olds were the largest age group (21.4%). White, Non-Hispanics constituted the most frequent race/ethnic group. Although, race/ethnicity was categorized as other or unknown by only 1.9% of the patients, they represented 2.3% of the patients in the PHX study area, compared to 1.4% outside the study area. Nearly half of the non-MVC trauma patients in AZ had at least one pre-injury chronic disease at the time of their injury (49.2%). Overall, chronic diseases were more prevalent among the study patients, 65.2%, versus 42.3% among non-MVC trauma patients living outside the study area. One noteworthy exception to this trend is alcoholism which is more prevalent outside the study area, however the difference is modest, 7.9% outside the study area, compared to 5.6% within the greater PHX area. Although ‘Do Not Resuscitate’ orders are not a chronic disease process, per se, such orders usually reflect some underlying level of acute or chronic condition. Do Not Resuscitate orders accompanied only a small proportion of the overall group (2.0%) though the hospital mortality rate among these patients was the highest among other chronic conditions (9.0%). Nearly 90% of the patients in this study had some form of insurance, though a greater proportion of the PHX area patients had no insurance. Table 1.

**Table 1:** Demographic Characteristics of 117,253 Non-MVC trauma patients, Arizona 2013–2017*

	All Patients	PHX	Outside PHX
N, (%)	117,253	63,451 (54.0)	53,802 (45.9)
Age Group, years, n (%)			
Younger than 5	5948 (5.1)	3637 (5.7)	2311 (4.3)
5–19	13,118 (11.2)	6342 (10.0)	6776 (12.6)
20–29	13,722 (11.7)	6558 (10.3)	7164 (13.3)
30–39	11,685 (10.0)	5501 (8.7)	6184 (11.5)
40–49	10,167 (8.7)	5058 (8.0)	5109 (9.5)
50–69	25,297 (21.6)	13,440 (21.2)	11,857 (22.0)
70–84	23,187 (19.8)	13,592 (21.4)	9595 (17.8)
85 and older	14,127 (12.1)	9323 (14.7)	4804 (8.9)
Sex, n (%)			
Male	68,885 (58.8)	35,598 (56.1)	33,287 (61.9)
Female	48,359 (41.2)	27,852 (43.9)	20,507 (38.1)
Race/Ethnicity, n (%)			
White	77,731 (66.3)	45,070 (71.0)	32,661 (60.7)
Hispanic/Latino, Any Race	20,144 (17.2)	10,972 (17.3)	9172 (17.1)
American Indian	12,029 (10.3)	2141 (3.4)	9888 (18.4)
Black	4060 (3.5)	3104 (4.9)	956 (1.8)
Asian	861 (0.7)	597 (0.9)	264 (0.5)
Hawaiian/Pacific Islander	197 (0.2)	113 (0.2)	84 (0.2)
Other/Unknown	2231 (1.9)	1454 (2.3)	777 (1.4)
Pre-Injury Chronic Diseases, n (%)			
One or more chronic diseases	64.101 (49.2)	41,368 (65.2)	22,733 (42.3)
Hypertension	31,807 (27.1)	21,857 (34.5)	9950 (18.5)
Smoker	14,058 (12.0)	8647 (13.6)	5411 (10.1)
Diabetes	13,225 (11.3)	8733 (13.8)	4492 (8.4)
Alcoholism	7828 (6.7)	3565 (5.6)	4263 (7.9)
Respiratory Disease	6467 (5.5)	4310 (6.8)	2157 (4.0)
Psychiatric Disorder	5958 (5.1)	4072 (6.4)	1886 (3.5)
Blood Disorder	5602 (4.8)	4509 (7.1)	1093 (2.0)
Dementia	5537 (4.7)	4230 (6.7)	1307 (2.4)
Congestive Heart Failure	3548 (3.0)	2624 (4.1)	924 (1.7)
Do Not Resuscitate Status	2289 (2.0)	1783 (2.8)	506 (0.9)
Insurance Status, n (%)			
Medicaid/Medicare/Government	72,626 (61.9)	37,666 (59.4)	34,960 (65.0)
Private/Commercial	28,896 (24.6)	17,248 (27.2)	11,648 (21.7)
Uninsured	9187 (7.8)	5231 (8.2)	3956 (7.4)
Other/Unknown	3910 (3.3)	1891 (3.0)	2019 (3.8)
Worker’s Compensation	2572 (2.2)	1395 (2.2)	1177 (2.2)
Vehicle Policy	62 (0.1)	–	42 (0.1)
Year of Injury			
2013	18,421 (15.7)	9247 (14.6)	9174 (17.1)
2014	21,220 (18.1)	11,207 (17.7)	10,013 (18.6)
2015	23,369 (19.9)	12,750 (20.1)	10,619 (19.7)
2016	25,309 (21.6)	14,239 (22.4)	11,070 (20.6)
2017	28,934 (24.7)	16,008 (25.2)	12,926 (24.0)
Died	2781 (2.4)	1704 (2.7)	1077 (2.0)

* *p* < 0.001 for all comparisons

In terms of the trauma events themselves, the vast majority were considered to be unintentional (91.1%) and 82.6% were due to blunt mechanisms of injury. More patients required care for injuries related to falls than any other mechanism (40,701, or 77.7% of all trauma patients in the study area). As a group, penetrating injury mechanisms were far less common (9153 [5.2%]) compared to the blunt mechanisms over the period of the study. Firearm and self-harm had the highest hospital mortality rates, 17.7 and 25.7%, respectively. When firearms were involved with the intention of self harm, the mortality exceeded 50% (*n* = 717 trauma events with 387 deaths [54.0%]). Most (61%) patients were transported by ground ambulance. Helicopter transport was employed for only 2.2% of the events, including 147 (8.6%) fatalities. However, patients transported by air had higher average probability of death from anatomic injuries (0.081 probability of dying from their injuries, sd 0.165), compared to patients transported by ground ambulance (0.033, sd 0.100). The majority of study patients (67.0%) were treated at Level I Trauma Centers and 87.9% of the fatalities were associated with Level I Trauma Centers. Of the study patients seen at Level I Trauma Centers, 3.5% died. Patients were primarily admitted to the hospital ward (39.4%) followed by being discharged to home (20.2%). Table 2.

**Table 2:** Trauma mechanism and mortality, 63,451 patients, Greater Phoenix, AZ 2013–2017*

	All Patients	Survived	Died
Injury Intent: n (%)			
Unintentional	47,730 (91.1)	46,933 (91.1)	797 (91.0)
Assaults	4422 (8.4)	4357 (8.5)	65 (7.4)
Undetermined	93 (0.2)	89 (0.2)	–
Self-harm	172 (0.3)	162 (0.3)	–
Mechanism of Injury, n (%)			
Blunt mechanisms			
Fall	40,701 (77.7)	40,000 (77.6)	701 (80.0)
Struck by, against	6485 (12.4)	6406 (12.4)	79 (9.0)
Transport, other	2182 (4.2)	2145 (4.2)	37 (4.2)
Pedal cycle related	1763 (3.4)	1740 (3.4)	–
Pedestrian related	844 (1.6)	809 (1.6)	35 (4.0)
Machinery	442 (0.8)	441 (0.9)	–
Penetrating			
Firearm	2874 (53.0)	2317 (48.0)	557 (93.6)
Cut/Pierce	2549 (47.0)	2511 (52.0)	38 (6.4)
Not recorded	5458 (8.6)	5257 (8.5)	201 (11.8)
Transport mode, n (%)			
Ground ambulance	48,208 (76.0)	46,705 (75.6)	1503 (88.2)
Private vehicle	13,462 (21.2)	13,416 (21.7)	46 (2.7)
Helicopter ambulance	1422 (2.2)	1275 (2.1)	147 (8.6)
Police vehicle	146 (0.2)	145 (0.2)	–
Other/unknown	200 (0.3)	194 (0.3)	–
Trauma Center Level, n (%)			
Level I	42,492 (67.0)	40,994 (66.4)	1498 (87.9)
Level III	13,181 (20.8)	13,077 (21.2)	104 (6.1)
Level IV	4665 (7.4)	4635 (7.5)	30 (1.8)
Non-Designated	3070 (4.8)	2998 (4.9)	72 (4.2)
TMPM Probability of Death,^a^	0.01 (0.01)	0.01 (0.01)	0.14 (0.46)
Destination From Emergency Department, n (%)			
Admitted to Hospital Ward	29,173 (46.0)	29,025 (47.1)	148 (8.7)
Home	12,821 (20.2)	12,821 (20.8)	–
Intensive Care Unit	10,071 (15.9)	9386 (15.2)	685 (40.2)
Unknown/Omitted Status	2882 (4.5)	2811 (4.6)	71 (4.2)
Operating Room	4232 (6.7)	4026 (6.5)	206 (12.1)
Eloped from ED	227 (0.4)	227 (0.4)	–
Transfer to Higher Level of Care	3451 (5.4)	3451 (5.6)	
Died in ED	592 (0.9)		

* *p* < 0.001 for all comparisons

-- denotes cell value < 25 persons

^a^ median (IQR)

The predictors of interest, population density, alcohol outlet density, ADI, the percentage of non-White race, and the percentage of urban population all demonstrated clustered patterns of autocorrelation. Of these, ADI demonstrated the greatest degree of clustering (Moran’s I = 0.63, *z* = 55.2), while alcohol outlet density was least clustered (Moran’s I = 0.08, *z* = 8.7). Table 3.

**Table 3:** Global and local spatial autocorrelation of predictors and types of trauma in Greater Phoenix

	Global			LISA Clusters			
Parameter	Moran’s I	Z score	HH	LL	LH	HL	*p* < 0.05*
Predictors							
Population/sq. mi	0.39	31.3	177	520	35	24	377
Alcohol Outlets/10,000 Pop.	0.08	8.7	78	95	114	16	62
Areal Deprivation Index	0.63	55.2	545	353	12	23	523
Percent Non-White Race Population	0.37	29.8	256	365	71	41	354
Percent Urban	0.49	38.3	350	68	1	80	441
Outcomes							
Non-Motor Vehicle Trauma/10,000	−0.02	−1.73	2	106	113	41	163
Assault-Related Trauma/1000	−0.02	−1.17	2	16	134	81	124

* Number of clusters with *p* < 0.005 for LISA

### Non-MVC trauma

**Fig. 3:**
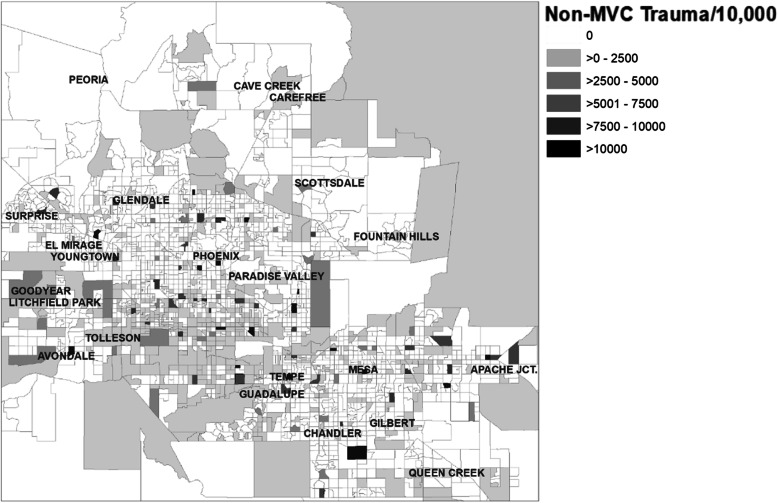
Geographic distribution of non-MVC traumas events, Greater Phoenix Area

The distribution of non-MVC trauma events per block group is shown in Fig. 3. The rate of non-motor vehicle trauma events per 10,000 population demonstrated a negative Moran’s I statistic and thus a dispersed autocorrelation pattern (− 0.02, *z* = 11.73). Furthermore, the LISA results (2 HH clusters, 106 LL clusters, 113 LH outliers, and 41 HL outliers) also depict a dispersed distribution of events. Table 3.

The GWR model for the rate of non-MVC trauma events per 10,000 population accounted for population density, ADI, the density of alcohol retail outlets, percent non-White race population, and percent urbanity. Three significant predictors emerged, population density, alcohol outlet density, and ADI. It is worth noting that the although effect size of population density is small (min − 0.08, max − 0.07, *p* < 0.01 in all block groups), the population density ranges from 0 to 39,300.3 persons per sq. mi.

Alcohol outlet density was a significant predictor of non-MVC trauma events in 499 block groups (19.9% of all block groups in the study area) or an area of 1475 sq.mi where over 800,000 Arizonans resided. In the block groups where the effect of alcohol outlet density was significant, the effect size ranged from 0.05 to 0.57 with a mean coefficient of 0.188. In those same areas, the mean alcohol outlet density was 8.97 per 10,000 population (95% Confidence Interval [CI] 7.41 to 10.53). As such, there are areas where alcohol outlet density increased the likelihood of non-MVC trauma events substantially, while in the majority of the study area, it had no significant effect.

ADI increased the likelihood of non-MVC trauma events across the region with a roughly uniform effect size, (mean coefficient 0.048, 95%CI 0.048 to 0.049). Of note, the model we report for areal associations with non-MVC trauma events demonstrates very poor fit to the data, accounting for only 1% of the variance in the phenomenon of interest Table 4.

**Table 4:** GWR model for non-MVC-related trauma events

Parameters	Model Coefficients		P-values			
	min	max	mean	min	max	mean
Population Density	−0.077	− 0.068	− 0.071	0.001	0.005	0.003
Alcohol Outlets Density	−0.002	0.566	0.109	< 0.001	0.99	0.30
Areal Deprivation Index	0.047	0.052	0.048	0.03	0.05	0.04
Percent Non-White Race	−0.003	0.023	0.016	0.32	0.99	0.51
Percent Urbanity	0.004	0.018	0.010	0.45	0.88	0.68

Local *R*^2^: min 0.01, max 0.01, mean 0.01

The residuals from the GWR model indicate locations where the number of observed non-MVC events were greater or lesser than predicted values for each location by the GWR model. This shows numerous “hot spot” areas contrasting from the surrounding areasFig. 4.

**Fig. 4:**
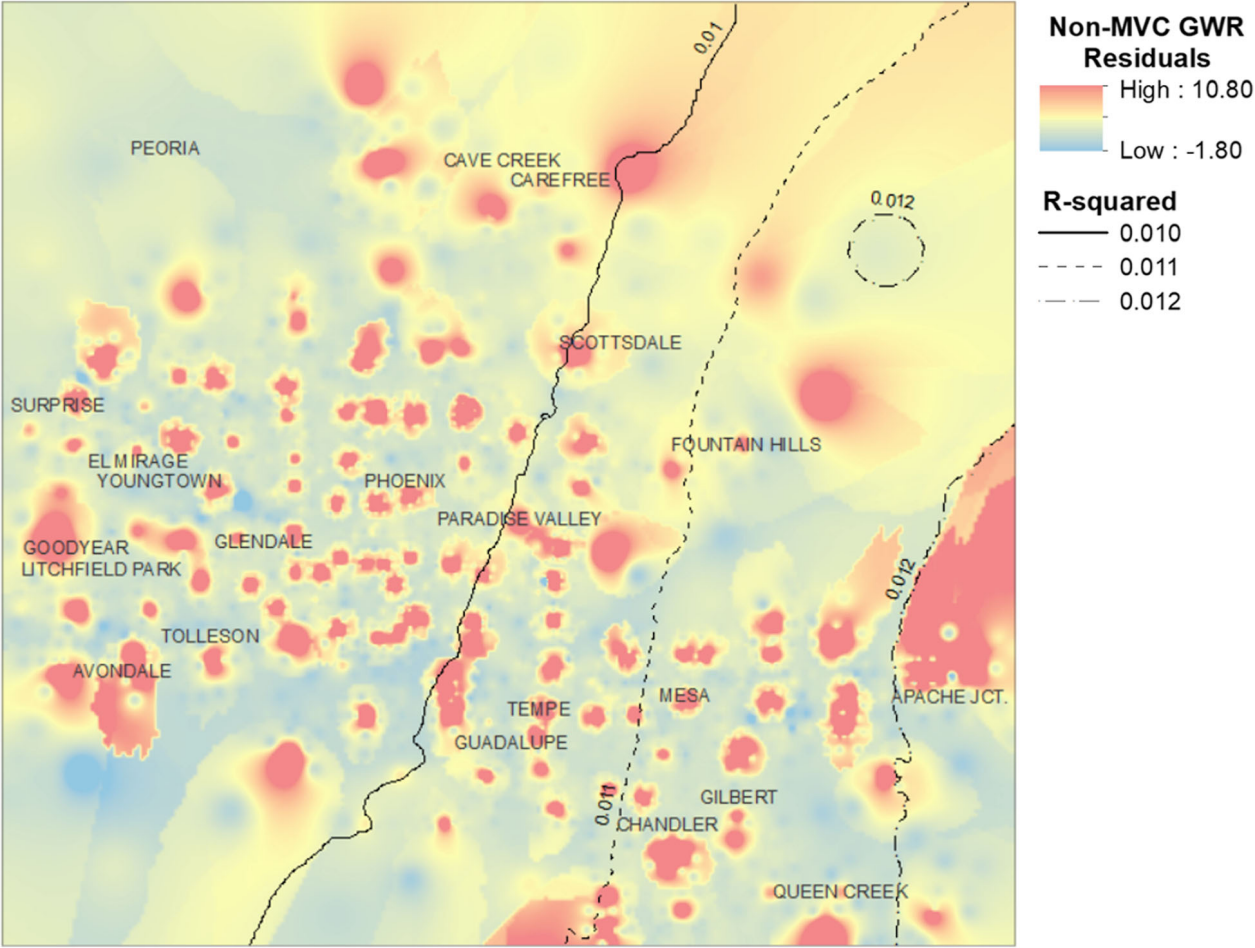
GWR model residuals for non-MVC trauma events, Greater Phoenix Area

### Assault-related trauma

**Fig. 5:**
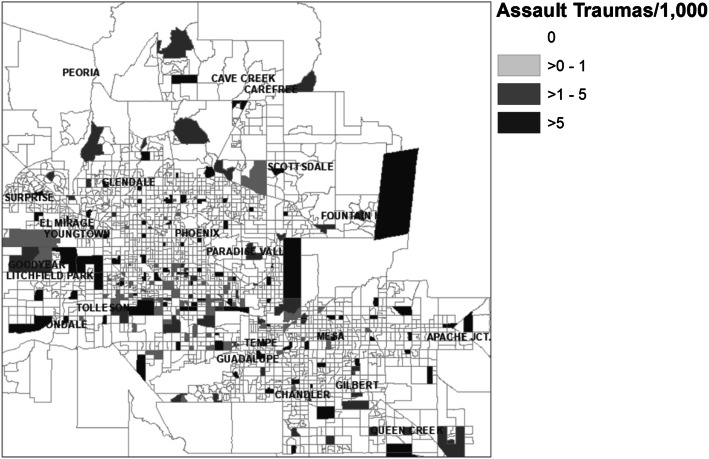
Geographic distribution of assaultive traumas events, Greater Phoenix Area

There were 7185 patients transported and treated for injuries sustained from being assaulted. Of these 388 (5.4%) died from their wounds. Assaultive injury events took place in 222 block groups and covering only 276 sq. mi. (7.3%) of the study area. A choropleth map showing the geographic distribution of these events is shown in Fig. 5.

Assaults are a proper subset (11.3%) of non-MVC trauma events and both types of injury events demonstrated dispersed patterns of autocorrelation. See Table 3.

Several characteristics distinguished the group of study patients injured by assault compared to the larger group of study patients comprised of all other non-MVC mechanisms of injury. These differences include age groups, sex, race, insurance status, and proportion over time. In contrast to the non-assault group where the most frequently treated patients were 70–84 years old (24%), the largest age group of assaulted patients age were 20–29 years old (33.8%). Males represented a larger proportion of assault victims than the proportion of males in the non-assault patient group (84.1% vs. 52.5%, respectively). Assault and non-assault patient differed by the relative proportions of race/ethnicity groups. People of Hispanic/Latino ethnicity of any race, American Indians, and Blacks represented greater proportions of assaultive traumas compared to the non-assaulted group among whom Whites were the vast majority. The proportion of American Indians in the assaultive trauma group is nearly four times the proportion of non-assaulted American Indians (10.4% vs. 2.5%), twice the proportion of American Indians in AZ (5.3%), and eight times the proportion in the United States (1.3%).(United States Census Bureau, 2018b) One quarter of the assaultive trauma patients were uninsured compared to only 6.2% of the non-assault group. See Table 5.

**Table 5:** Comparison of assault to non-assault patients, Greater Phoenix, AZ*

	Assaults	Non-Assaults
N, (%)	7185 (11.3)	56,266 (88.7)
Age Group, years, n (%)		
Younger than 5	16 (0.2)	3621 (6.4)
5–19	756 (10.5)	5586 (9.9)
20–29	2427 (33.8)	4131 (7.3)
30–39	1747 (24.3)	3754 (6.7)
40–49	1147 (16.0)	3911 (7.0)
50–69	1001 (14.0)	12,439 (22.1)
70–84	71 (1.0)	13.521 (24.0)
85 and older	20 (0.3)	9303 (16.5)
Sex, n (%)		
Male	6044 (84.1)	29,554 (52.5)
Female	1140 (15.9)	26,712 (47.5)
Race/Ethnicity, n (%)		
White	2993 (41.7)	42,077 (74.8)
Hispanic/Latino, Any Race	2014 (28.0)	8958 (15.9)
American Indian	744 (10.4)	1397 (2.5)
Black	1077 (15.0)	2027 (3.6)
Asian	63 (0.9)	534 (1.0)
Hawaiian/Pacific Islander	–	89 (0.2)
Other/Unknown	270 (3.8)	1184 (2.1)
Pre-Injury Chronic Diseases, n (%)		
One or more chronic diseases	3678 (51.2)	37,690 (67.0)
Insurance Status, n (%)		
Medicaid/Medicare/Government	3779 (52.6)	33,887 (60.2)
Private/Commercial	1303 (18.1)	15,945 (28.3)
Uninsured	1758 (24.5)	3473 (6.2)
Other/Unknown	279 (3.9)	1612 (2.9)
Worker’s Compensation	65 (0.9)	1330 (2.4)
Vehicle Policy	–	–
Year of Injury		
2013	1350 (18.8)	7897 (14.0)
2014	1282 (17.8)	9925 (17.6)
2015	1437 (20.0)	11,313 (20.1)
2016	1539 (21.4)	12,700 (22.6)
2017	1577 (22.0)	14,431 (25.7)
Died	388 (5.4)	1316 (2.3)

* *p* < 0.001 for all comparisons

-- denotes cell value < 25 persons

Assaultive trauma event locations were modeled for association with the hypothesized predictors using GWR. Alcohol outlet density was a significant, positive, independent predictor of assaultive events in 132 block groups. In those block groups, the mean coefficient for alcohol outlet density was 0.102 (95% CI 0.100 to 0.104, min 0.065, max 0.120) and the mean alcohol outlet density (n outlets/10,000 people) was 11.28 (95% CI 8.18 to 11.37, min 0, max 100.14). ADI was a positive predictor with a near uniform effect size of 0.065 in all block groups. While the coefficient of regression exhibited very little nonstationarity, the ADI ranged from 2 to 148 in the study area.

Population density was a statistically significant, negative predictor of the locations of assaultive trauma with a mean coefficient of − 0.060 across the study area where the population ranged from 0 to 39,300.3 persons per square mile. The percentage non-White race increased the likelihood of assaultive trauma events by a mean coefficient of 0.07 (95% CI 0.070 to 0.072, min 0.033, max 0.090). Table 6. The association of percent non-White race and location of assaultive trauma was statistically significant in 77.4% of census block groups with 75.1% of the study area population. Percent urban population was a significant predictor of assaultive trauma events in 218 block groups which accounts for 51.2 sq. mi. or 1.4% of the study area and 7.8% of the study area population. The mean population density in these areas was 7450.8 per sq. mi. (95% CI 6834.0 to 8067.5), whereas the mean population density in areas where the effect of percent urbanity was not significant was 5026.1 per sq. mi. (95% CI 4872.8 to 5179.5). The local R^2^ values for this model ranged from 0.01 to 0.02 indicating the model accounts for only 1–2% of the variation of assaultive trauma events in PHX. Table 6.

**Table 6:** GWR model for assault-related trauma events

Parameters	Local Model Coefficients			Local P-values		
	min	max	mean	min	max	mean
Population Density	−0.063	−0.054	−0.060	0.007	0.025	0.011
Alcohol Outlets Density	0.001	0.120	0.051	0.026	0.979	0.317
Areal Deprivation Index	0.059	0.069	0.065	0.004	0.017	0.006
Percent Non-White Race	0.033	0.090	0.071	< 0.001	0.219	0.028
Percent Urbanity	−0.175	0.571	0.046	0.001	> 0.99	0.509

Local *R*^2^: min 0.01 max 0.02 mean 0.02

**Fig. 6:**
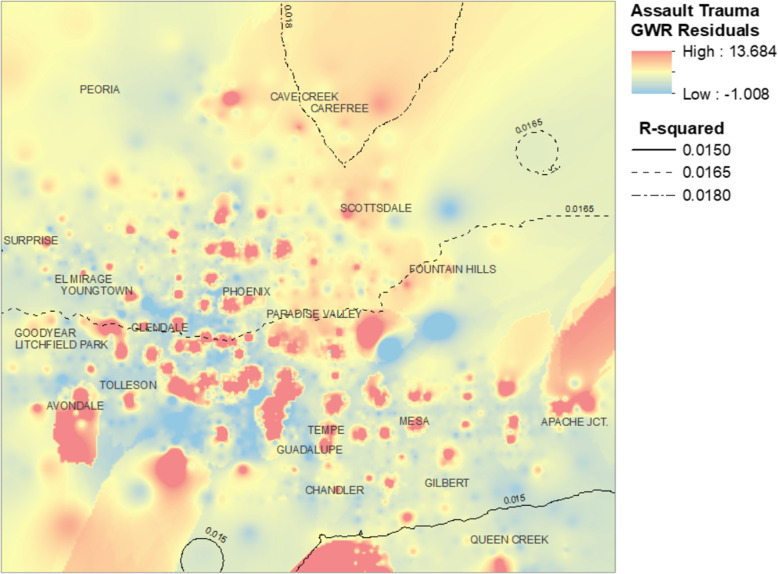
GWR model residuals for assaultive trauma events, Greater Phoenix Area

Fig. 6 shows the residuals from the GWR regression model for assault-related trauma events plotted over the study area. Like the residual map for non-MVC trauma there are numerous areas of positive residual assaultive trauma events. However, in contrast to Fig. 4, the hot spots appear to be more concentrated in the areas of Glendale, central and southern Phoenix, Paradise Valley, and Tempe.

## Discussion

Arizona has several unique characteristics which represent opportunities to refine the magnitude of known associations of areal sociodemographic features with the occurrence and locations of trauma. For example, 17.9% of the AZ population lives below the poverty level compared to 15.4% for the overall US. American Indians and Arizonans of Hispanic/Latino(a) ethnicity represent 4.4 and 29.9% of the Arizona population, respectively, whereas in the general US population, they constitute 0.8 and 16.6%, respectively.(United States Census Bureau, 2015) The associations we observed between the occurrence of traumatic injuries and the environmental features where they occur, specifically SES and alcohol availability demonstrate nonstationary associations with non-MVC trauma and assaultive trauma events.

### Alcohol outlet density

Alcohol outlet density was found to have positive, albeit modest, associations with non-MVC traumas per 10,000 population and assault-related trauma events per 1000 population in portions of the greater PHX area when population density, percent non-White population, and percent urban population were accounted for in the model. Alcohol outlet density was a significant predictor of non-MVC trauma events in the block groups where approximately 20% of the population in the study area lives, with other areas left unaffected. The density of alcohol outlets was associated with assault-related trauma events in block groups where 5% of the population lived. Then, only 2.9% of the population lived in block groups where alcohol outlet density was associated with both non-MVC and assaultive trauma event locations.

The preponderance of the literature pertaining to alcohol outlet density and traumatic injury finds significant, positive associations between these phenomena. For example, Mair, et al., found a significant and positive association between alcohol outlet densities, defined as the number of outlets per square mile, and violent assaults in a statewide analysis of California, with relative risks ranging from 1.018 to 1.023.(Mair et al., 2012) LaScala, et al., defined as outlets per kilometer of roadway per census tract in San Francisco County, California among hypothesized predictors of pedestrian injuries.(LaScala et al., 2001) They differentiated outlets as bars, restaurants or off-premise outlets. The off-premise outlets carried no association with their outcome of interest.(LaScala et al., 2001) However, in a study limited to New York City, DiMaggio found that census tracts with one or more alcohol retail outlets were at 47% increased risk for bicyclist or pedestrian injuries.(DiMaggio et al., 2016)

### Socioeconomic deprivation

Trauma is an equal opportunity disease that occurs throughout AZ, across age and racial/ethnic groups and levels of SES. In the areas where ADI was statistically significant, every 20-point increase in ADI was associated with an increase of one non-MVC trauma event per 10,000 persons. Even in the absence of data regarding patients’ choices, attributes of the areas where traumas take place are distinguishable from places where traumas do not occur. Recently, Jarman, et al., analyzed data from the Maryland Adult Trauma Registry focusing on injury mortality. The authors classified regions based on areal characteristics at the Zip Code Tabulation Area level within the state. Eight categories based on mortality risk were constructed and included patient characteristics in addition to the areal attributes using a latent class analysis. They concluded that identifying patterns within the built environment interacting with patient characteristics can direct prevention efforts.(Jarman et al., 2018c) Cubbin, et al., studied the relationship between SES and the risk of injury by analyzing data from the National Health Interview Survey. They found SES to be a meaningful predictor if injury though the effect size was dependent on which measure of SES was applied as well as the cause and severity of traumatic injuries.(Cubbin et al., 2000a) Of note, the studies by Jarman, et al., and Cubbin, et al., did not include alcohol outlet location in the analysis.(Cubbin et al., 2000a; Jarman et al., 2018c) The present study is congruent with the studies by Jarman and Cubbin in finding that ADI was associated with the rate of non-MVC traumas per 10,000 population per block group in AZ even in the presence of alcohol retail outlet density and other confounders in the model.

### Population density

The effect of population density was significant and negative in both multivariable GWR models in this analysis. Population density has been explored as a predictor of myriad phenomena, including violent crimes and locations of traumatic injury with varying results. Prior to 1961, greater population density, particularly in urban areas, was considered an indicator of social disarray. Intuitively, the association of population density and violence may be thought of as linear and positive. However, Jacobs postulated that areas of population and dwelling concentration are vital to cities and not characteristic of social decline.(Jacobs, 1961) Instead, greater surveillance by virtue of a greater concentration of people was thought to be responsible for the lower degree of social disarray.(Jacobs, 1961) Cahill, et al., applied GWR to violent crime data for Portland, Oregon and found an inverse relationship of population density to local occurrences of crime.(Cahill & Mulligan, 2007) Lasecki, et al., investigated social factors associated with intentional injury in Mobile County, Alabama. Their study showed no association between population density and the risk for intentional injury.(Lasecki et al., 2018) More recently, Christens and Speer also demonstrated the negative association between population density and violent crime in Nashville, TN.(Christens & Speer, 2005) Similar negative associations between population density and non-intentional trauma events have also been observed in the Portland, OR area, and in Norway.(Feero et al., 1995; Kristiansen et al., 2014) The results observed in the present study adds to the evidence that population density is somewhat protective with regard to the incidents of serious traumatic injury.

### Percent non-white populations

Percent non-White race was significantly associated with assault-related trauma event locations across a majority of the study area. However, no such association was present with regard to the locations of non-MVC traumas. Interestingly, the percentage of non-White race was found to be a predictor of severe injury in a study by Newgard, et al., who analyzed data from nine study sites across the US and Canada.(Newgard et al., 2011) The effect of non-White population in their study was greatest in penetrating trauma mechanisms and intentional injuries.(Newgard et al., 2011) However, the process linking assaultive trauma and the percentage of non-White race population present in a region is unclear. It is also unclear how these factors interact with the behavior of individuals. We postulate that the phenomena captured by the variable percent non-White race in a block group’s population may actually reflect other areal or social characteristics associated with the occurrence of assaultive events.

### Limitations

The present study is subject to several limitations. First, the nature of the data analyzed in this study were not gathered for the purpose of a spatial analysis of trauma in AZ, the relationships of alcohol outlets to trauma and assaults, and ADI. Most importantly, the patient-level data originated as hospital records or trauma center registries. Thus, the expertise with which traumatic injury diagnoses are identified in the trauma registry or billing records can be highly variable one center to another. However, the data are vetted and cleaned by Arizona Department of Health Services, and represent the most complete record for AZ. Additionally, the ASTR data do not include trauma victims who were deceased at the scene and were transported to a morgue, thus bypassing the entry criteria for the registry. As such, this study does not allow for population-based inferences regarding all traumatic injury in the Greater PHX Area.

Next, the analysis relied on geocoded locations of traumatic injuries and alcohol outlets. Despite successfully mapping 88.3% of the trauma events, 23,581 cases were excluded due to missing location data, including 3877 assaults were unavailable for inclusion in the study. As a result, estimates of effect for alcohol outlet counts per block group, population density, and ADI may be biased as the pattern of missingness suggests correlation with other features rather than randomly occurring events. Delineating the underlying mechanisms enabling these patient characteristics to be associated with geocoded addresses is unknown and beyond the scope of this study, though may be a source of geographic bias.(Zimmerman, 2006) Geocoding errors have been shown to be more prevalent in rural areas, as is the case for roughly 10% of the state’s population (Hay et al., 2009; Arizona Office of Economic Opportunity, 2018).

Most importantly, the regression R^2^ values indicate the models capture only a small fraction of the variance of the phenomena of interest to the study. As such, the preponderance of predictors describing the rates of non-MVC and assaultive trauma events in the Greater PHX area were unmeasured by this study. It is also plausible that the overall dispersed nature of the outcomes of interest to this study preclude spatial association with geographically clustered predictors. Thus, the inferences of this study must be interpreted cautiously given these limitations.

## Conclusions

The locations of non-MVC trauma and assaultive injury events were geographically dispersed phenomena in Arizona. The associations with these trauma event locations with the hypothesized predictors of alcohol outlet density and ADI were present in a nonstationary fashion, though our model captured trace amounts of the variance in the greater PHX area. The risk factors for traumatic injury in Arizona communities are multifactorial, and represent both environmental hazards and individual choices. The effect of a particular promoter of traumatic injury in one local area may differ dramatically from its effect in neighboring areas. Therefore, interventions aimed at reducing the burden of trauma must be multidisciplinary, geographically targeted, and risk-specific. More investigation is warranted and needed to elucidate the injury-prone interactions of people and place, including the role of the racial and ethnic diversity.

## Data Availability

Please contact author for data requests.
